
JOURNAL INFORMATION
==============================
NLM Title Abbreviation: J Diabetes Investig
Journal ID: J Diabetes Investig
NLM Journal ID: 101520702
Journal ID: JDI

Journal of Diabetes Investigation

ISSN: 2040-1116
EISSN: 2040-1124
Publisher: Wiley

ARTICLE INFORMATION
==============================
PMCID: PMC11474508
DOI: 10.1111/jdi.14165
Article ID: JDI14165
Article version: 1
Subjects: Issue Information, Issue Information

Issue Information

Electronic publication date: 2024 Mar 1
Publication date: 2024 Mar
Volume: 15
Issue: 3
First page: 259
Last page: 260
Copyright: © 2024 The Authors. Journal of Diabetes Investigation published by Asian Association for the Study of Diabetes (AASD) and John Wiley & Sons Australia, Ltd
License: This is an open access article under the terms of the http://creativecommons.org/licenses/by/4.0/ License, which permits use, distribution and reproduction in any medium, provided the original work is properly cited.
License URL: https://creativecommons.org/licenses/by/4.0/

Figure count: 0
Table count: 0
Page count: 5

source-schema-version-number: 2.0
cover-date: March 2024
details-of-publishers-convertor: Converter:WILEY_ML3GV2_TO_JATSPMC version:6.4.9 mode:remove_FC converted:15.10.2024

==============================

### JDI

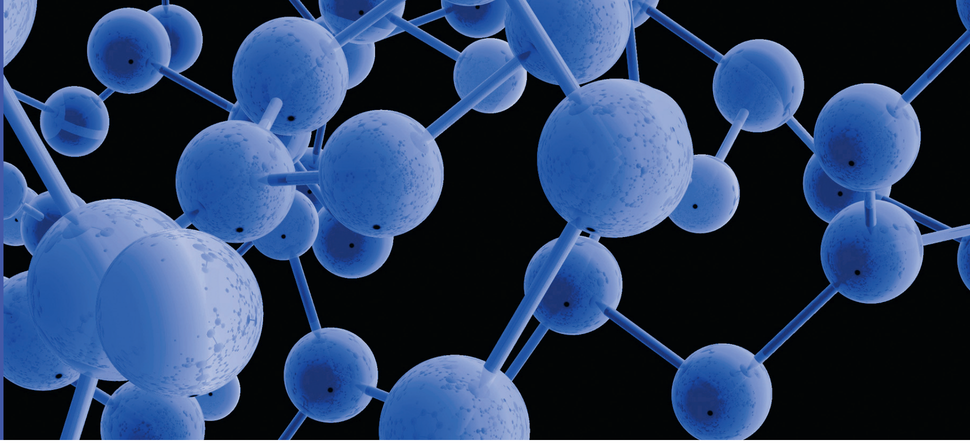

Volume 15 | Issue 3
March 2024

### Journal of Diabetes Investigation

Official Journal of the Asian Association for the Study of Diabetes

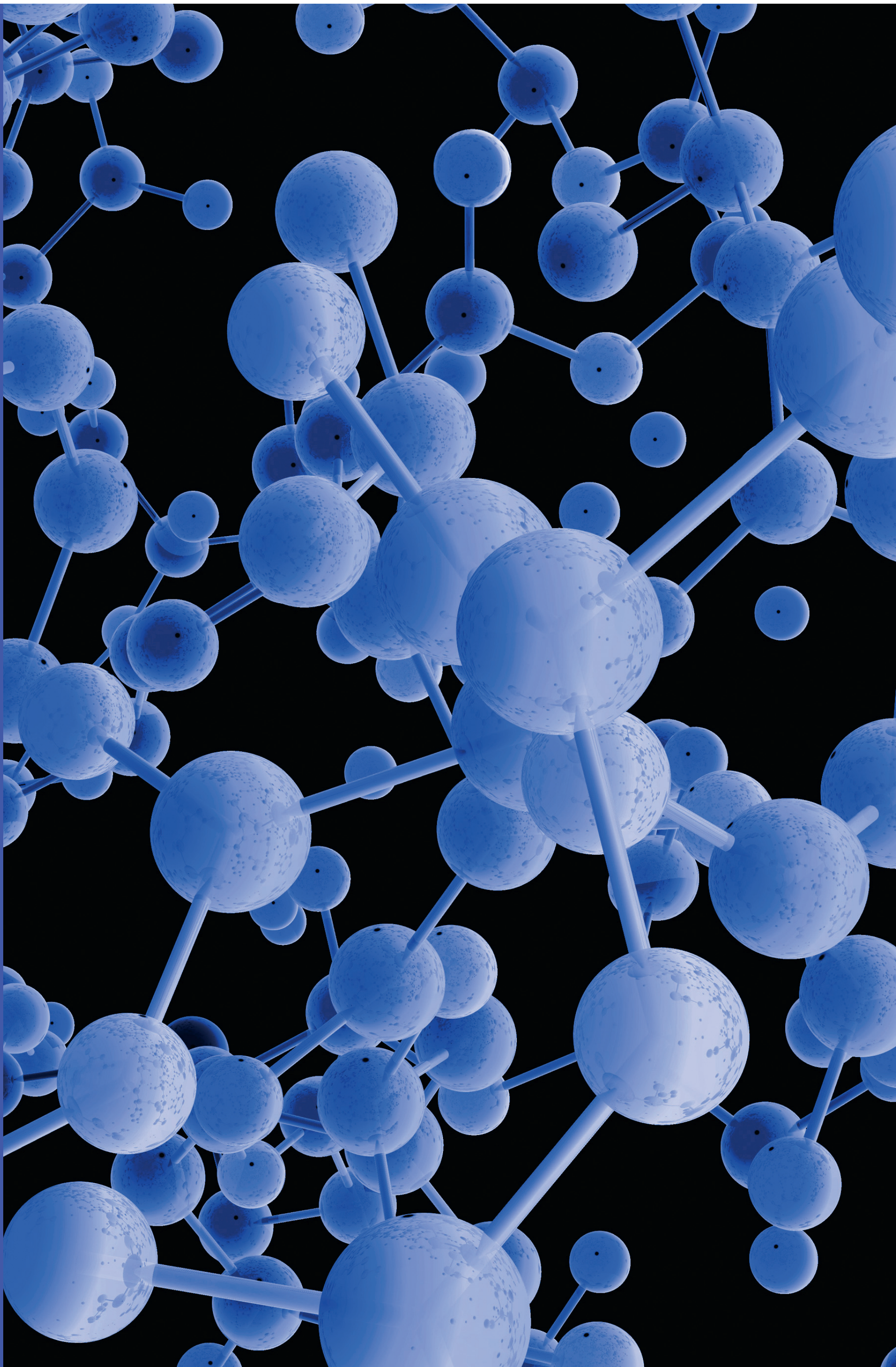

HCRU and costs of SGLT2i vs DPP4i

THE ASIAN ASSOCIATION FOR THE STUDY OF DIABETES

WILEY

JDI Journal of Diabetes Investigation
Official Journal of the Asian Association for the Study of Diabetes
Open access

**Editor-in-Chief**
Nigishi Hotta, *Japan Organization of Occupational Health and Safety, Chubu Rosai Hospital, Nagoya, Japan*

**Co-Editor**
Domenico Accili, *Columbia University, New York, USA*

**Expert Editor**
Bo Ahren, *Lund, Sweden*
Young Min Cho, *Seoul, Korea*
Sean Dinneen, *Galway, Ireland*
Marc Gilbert, *Montpelier, France*
Limei Liu, *Shanghai, China*
Ronald Ma, *Hong Kong, China*
Louis Philipson, *Chicago, USA*
Wayne H-H Sheu, *Taichung, Taiwan*
Bernard Thorens, *Lausanne, Switzerland*
Daisuke Yabe, *Gifu, Japan*

**Editorial Board**
Eiichi Araki, *Kumamoto, Japan*
Xiaoping Chen, *Beijing, China*
Lee-Ming Chuang, *Taipei, Taiwan*
Masakazu Haneda, *Asahikawa, Japan*
Larry Low-Tone Ho, *Taipei, Taiwan*
Tianpei Hong, *Beijing, China*
Akihisa Imagawa, *Osaka, Japan*
Nobuya Inagaki, *Kyoto, Japan*
Linong Ji, *Beijing, China*
Weiping Jia, *Shanghai, China*
Yi-Der Jiang, *Taipei, Taiwan*
Takashi Kadowaki, *Tokyo, Japan*
Kohei Kaku, *Kurashiki, Japan*
Keizo Kanasaki, *Izumo, Japan*
Doo-Man Kim, *Seoul, Korea*
Karen Lam, *Hong Kong, China*
Hong Kyu Lee, *Gyeonggi-do, Korea*
In-Kyu Lee, *Daegu, Korea*
Moon-Kyu Lee, *Seoul, Korea*
Jiro Nakamura, *Nagakute, Japan*
Kishio Nanjo, *Wakayama, Japan*
Wataru Ogawa, *Kobe, Japan*
Yutaka Seino, *Osaka, Japan*
Shyi-Jang Shin, *Kaohsiung, Taiwan*
Hirohito Sone, *Niigata, Japan*
Kathryn Tan, *Hong Kong, China*
Yukio Tanizawa, *Ube, Japan*
Kohjiro Ueki, *Tokyo, Japan*
Jun Wada, *Okayama, Japan*
Hirotaka Watada, *Tokyo, Japan*
Jianping Weng, *Guangzhou, China*
Soroku Yagihashi, *Hirosaki, Japan*
Hiroshi Yamamoto, *Kanazawa, Japan*
Toshimasa Yamauchi, *Tokyo, Japan*
Wenying Yang, *Beijing, China*
Kazuki Yasuda, *Tokyo, Japan*
Koutaro Yokote, *Chiba, Japan*
Kun-Ho Yoon, *Seoul, Korea*
Dalong Zhu, *Nanjing, China*

**International Editorial Board**
Michael A. Brownlee, *New York, USA*
Subrata Chakrabarti, *Ontario, Canada*
Mark E. Cooper, *Prahran, Australia*
Philippe Froguel, *London, UK*
Wilfred Fujimoto, *Hawaii, USA*
Michael S. German, *San Francisco, USA*
Andrew Hattersley, *Exeter, UK*
Jean-Claude Henquin, *Brussels, Belgium*
Jens Juul Holst, *Copenhagen, Denmark*
Frank B. Hu, *Boston, USA*
C. Ronald Kahn, *Boston, USA*
George L. King, *Boston, USA*
Rayaz A. Malik, *Manchester, UK*
Michael Mauer, *Minneapolis, USA*
Carl Erik Mogensen, *Aarhus, Denmark*
Steven E. Shoelson, *Boston, USA*
Jay S. Skyler, *Miami, USA*
Paul Zimmet, *Melbourne, Australia*

**Assistant Editorial Board**
Tien-Jyun Chang, *Taipei, Taiwan*
Yoshiyuki Hamamoto, *Osaka, Japan*
Norio Harada, *Kyoto, Japan*
Yi-Jen Hung, *Taipei, Taiwan*
Chi-Min Hwu, *Taipei, Taiwan*
Hideki Kamiya, *Aichi, Japan*
Dae Jung Kim, *Suwon, Korea*
Seung-Hwan Lee, *Seoul, Korea*
Hung-Yuan Li, *Taipei, Taiwan*
Takashi Matsuzaka, *Tsukuba, Japan*
Keiko Naruse, *Aichi, Japan*
Takeshi Nishikawa, *Kumamoto, Japan*
Eung-Jung Rhee, *Seoul, Korea*
Yusuke Seino, *Aichi, Japan*
Hironori Waki, *Tokyo, Japan*

**Aims and Scope:** *Journal of Diabetes Investigation* is your core diabetes journal from Asia; the official journal of the Asian Association for the Study of Diabetes (AASD). The journal publishes original research, country reports, commentaries, reviews, mini-reviews, case reports, letters, as well as editorials and news. The journal embraces clinical and experimental research in diabetes and related areas. This includes aspects of prevention, treatment, as well as molecular aspects and pathophysiology. Translational research focused on the exchange of ideas between clinicians and researchers is also welcome.

**Cover:** Insulin molecule © Pasieka/Science Photo Library

This journal is available online at Wiley Online Library. Visit wileyonlinelibrary.com to search the articles and register for table of contents and e-mail alerts.

**For submission instructions, and all other information visit wileyonlinelibrary.com/journal/jdi**

JDI.JEB.Mar24

**Open Access and Copyright**

All articles published by *Journal of Diabetes Investigation* are fully open access: immediately freely available to read, download and share. All *Journal of Diabetes Investigation* articles accepted from 24 September 2013 are published under the terms of the Creative Commons Attribution 4.0 Unported (CC BY 4.0) license, which permits use, distribution and reproduction in any medium, provided the original work is properly cited. Articles accepted before this date were published under the agreement as stated in the final article. Copyright on any *Journal of Diabetes Investigation* article accepted after 24 September 2013, the date on which *Journal of Diabetes Investigation* became an open access journal, is retained by the author(s). Authors grant to *Journal of Diabetes Investigation* and Wiley a license to publish the article and for Wiley to identify itself as the original publisher. Authors also grant any third party the right to use the article freely as long as its integrity is maintained and its original authors, citation details and publisher are identified. Further information about open access licenses and copyright can be found at: https://authorservices.wiley.com/author-resources/Journal-Authors/licensing/licensing-info-faqs.html#4. Information on the licenses used by Journal of Diabetes Investigation can be found at: wileyonlinelibrary.com/journal/jdi

**Disclaimer**

The Publisher, the Asian Association for the Study of Diabetes, and Editors cannot be held responsible for any errors in or any consequences arising from the use of information contained in this journal. The views and opinions expressed do not necessarily reflect those of the Publisher, the Asian Association for the Study of Diabetes or Editors, neither does the publication of advertisements constitute any endorsement by the Publisher, the Asian Association for the Study of Diabetes, Editors, or Authors, of the products advertised.

Wiley Open Access articles posted to repositories or websites are without warranty from Wiley of any kind, either express or implied, including, but not limited to, warranties of merchantability, fitness for a particular purpose, or non-infringement. To the fullest extent permitted by law Wiley disclaims all liability for any loss or damage arising out of, or in connection, with the use of or inability to use the content.

JDI.PI.Mar24

VOLUME 15 – ISSUE 3 – MARCH 2024

### Journal of Diabetes Investigation

Open access

### Review

**Overnutrition in the early postnatal period influences lifetime metabolic risk: Evidence for impact on pancreatic β-cell mass and function**
Joshua JN Burton and Laura C Alonso **263**

### Mini Review

**Recurrent hypoglycemia induced by clopidogrel: A case report and mini review**
Weiguan Du, Haining Wang, Ye Liu, and Tianpei Hong **275**

### Commentary

**Glucose-dependent insulinotropic polypeptide receptor systems in the hypothalamus and the brainstem regulate feeding and weight *through* distinct pathways**
Toshihiko Yada **282**

### Update

**Effectiveness and safety of sodium–glucose cotransporter 2 inhibitors in Asian populations**
Kyoung Hwa Ha and Dae Jung Kim **285**

### Original Articles

### Basic Science and Research

**METTL3/YTHDF2 m6A axis mediates the progression of diabetic nephropathy through epigenetically suppressing PINK1 and mitophagy**
Fangfang Wang, Juan Bai, Xin Zhang, Dali Wang, Xin Zhang, Jingwen Xue, Haoyang Chen, Shuxiang Wang, Baojin Chi, Jing Li, and Xiaoru Ma **288**

***Cordyceps cicadae* polysaccharides attenuate diabetic nephropathy *via* the miR-30a-3p/TRIM16 axis**
Rong Zheng, Qin Xu, Yiwen Wang, Yifei Zhong, and Rong Zhu **300**

### Clinical Science and Care

**Association between area under the C-peptide curve during an oral glucose tolerance test and diabetic retinopathy in type 2 diabetes patients**
Cong Zhang, Xuefei Ma, Wenjian Lin, Qian Xu, Hongxue Li, Chengye Xu, Ming Hao, and Hongyu Kuang **315**

**Prognostic value of longitudinal HbA1c variability in predicting the development of diabetic sensorimotor polyneuropathy among patients with type 2 diabetes mellitus: A prospective cohort observational study**
Yun-Ru Lai, Wen-Chan Chiu, Chih-Cheng Huang, Ben-Chung Cheng, I-Hsun Yu, Chia-Te Kung, Ting Yin Lin, Hui Ching Chiang, Chun-En Aurea Kuo, and Cheng-Hsien Lu **326**

**Effect of hemoglobin A1c management levels on coronary physiology evaluated by quantitative flow ratio in patients who underwent percutaneous coronary intervention**
Mingfeng Chen, Jichen Liu, Zhangxin Xie, Wei Chen, Yanqin Hu, Junping Wen, Jinyan Chen, Xuemei Chen, Lirong Lin, Rehua Wang, and Lihong Lu **336**

**Body composition and risk of gestational diabetes mellitus: A univariable and multivariable Mendelian randomization study**
Yuan Gao, Min Zhou, Xuan Xu, Jun Yang Ma, and Man Fen Qin **346**

**Lipid profile in women of different ethnicity with gestational diabetes: Relationship with fetal growth**
Maria Grazia Dalfrà, Silvia Burlina, Eugenio Ragazzi, Silvia Pastrolin, Giovanni Sartore, and Annunziata Lapolla **355**

**Type 1 diabetes and the risk of epilepsy: A meta-analysis**
Shuhui Wu and Yanan Ding **364**

**Healthcare resource utilization and healthcare costs in patients with type 2 diabetes mellitus initiating sodium-glucose cotransporter 2 inhibitors vs dipeptidyl peptidase-4 inhibitors in Japan: A real-world administrative database analysis**
Atsunori Kashiwagi, Shingo Shoji, Yoshinori Kosakai, Yoko Yoshinaga, and Mitsuhiro Rokuda **374**

### Case Report

**A case of type 2 diabetes mellitus with weight gain and worsening of glycemic management after tezepelumab administration for severe bronchial asthma**
Kotaro Umamoto, Ryotaro Bouchi, Noriko Ihana-Sugiyama, Noriko Kodani, Mitsuru Ohsugi, Masayuki Hojo, Kohjiro Ueki, and Hiroshi Kajio **388**
